# SiO_2_-CaO-P_2_O_5_ Bioactive Glasses: A Promising Curcuminoids Delivery System

**DOI:** 10.3390/ma9040290

**Published:** 2016-04-15

**Authors:** Valentina Nicolini, Monica Caselli, Erika Ferrari, Ledi Menabue, Gigliola Lusvardi, Monica Saladini, Gianluca Malavasi

**Affiliations:** Department of Chemical and Geological Sciences, University of Modena and Reggio Emilia, Via G. Campi 103, Modena 41125, Italy; valentina.nicolini@unimore.it (V.N.); monica.caselli@unimore.it (M.C.); erika.ferrari@unimore.it (E.F.); ledi.menabue@unimore.it (L.M.); gigliola.lusvardi@unimore.it (G.L.); monica.saladini@unimore.it (M.S.)

**Keywords:** bioactive glass, curcuminoids, drug release

## Abstract

In this paper, we report the study of the loading and the release of curcuminoids by bioactive glasses (BG) and mesoporous bioactive glasses (MBG). Through a detailed spectroscopic study, it was possible to determine the amount and the type of molecules released in water and in simulated body fluid (SBF). In particular, curcumin and K2T21 show a good ability to be released in di-keto and keto-enolic form, depending from the pH. However, after 24 h, the amount of pristine curcumin release is very low with a consequent increment of degradation products derived by curcuminoids. The presence of –OH groups on curcuminoids is a fundamental pre-requisite in order to obtain a high loading and release in polar solution such as water and SBF. The substrate on which we loaded the drugs does not seem to affect significantly the loading and the release of the drugs. The environment, instead, affects the release: for all the drugs, the release in SBF, buffered at pH of 7.4, is slightly worse than the release in water (basic pH values).

## 1. Introduction

Recent developments in bone tissue engineering provided alternative approaches for the repair of bone defects caused by trauma and infection [1]. Bone repair scaffolds loaded with drugs (*i.e.*, antibiotics and antitumoral medicaments) and/or growth factors, attract increasing attention since they can protect against infections but also regulate cell growth and enhance bone regeneration [2,3,4,5,6,7,8,9]. Basically, bone scaffolds should be biocompatible, biodegradable, osteoconductive and, in improved scaffold designs, they should be able to act as local drug carrier [2,9,10,11,12,13,14]. Scaffolds are usually made from tailored combination of inorganic and organic phases, forming composite structures aiming at replicate the structure and composition of bone tissue [2,3,13,15]. Several bioactive glasses and bioceramics have been used as the inorganic phase in drug eluting composite scaffolds, including hydroxyapatite (HA) [6,7,16], calcium phosphate (CaP) [17,18,19], and Bioglass^®^ [20,21,22,23]. Scaffolds composed of a single inorganic component usually have low drug binding affinity, and thus, they do not allow a controlled drug release [13]. This is particularly the case for bioactive glass scaffolds derived from molten glasses [20,21,22,23], which present a low specific surface area and do not have a suitable intrinsic porosity to be used as drug reservoirs [3,10]. Therefore, the sol-gel synthesis methodology yields high surface area materials. In particular, the sol-gel process coupled with *evaporation-induced self-assembly* (*EISA*) method allows preparing mesoporous bioactive glasses (MBG) (pore diameter: 2–10 nm) [22,24]. *In vitro*, these materials exhibit the quickest bioactive response among asynthetic materials. In addition, they display high surface areas and pore volumes, which are suitable features in order to host biologically active molecules/drugs [25,26,27]. In view of these characteristics, MBG systems can be considered good drug delivery system (DDS). The drug molecules can be hosted within the mesopores by soaking techniques and then released via a diffusion-based mechanism without drug-silica interaction [28]. However, the properties of the drugs (dimension and polarity) and the MBG surfaces (pore dimensions and functional group) could play an important role in the loading and releasing phases [29].

Between the drugs loaded on bioactive glasses, the antibacterial and anti-inflammatory substances received much attention. Recently, Malavasi *et al.*, tried to load curcumin on sol-gel (BG) and mesoporous bioactive glasses (MBG) [30,31] using immersion techniques, obtaining low amount of loaded drug: 0.4% wt of curcumin on powder glass.

Curcumin [1,7-bis(4-hydroxy-3-methoxyphenyl)-1,6-heptadiene-3,5-dione], commonly extracted from the dried rhizomes of Curcuma longa L., presents a wide spectrum of therapeutic properties, ranging from antioxidant and anti-inflammatory to chemo-preventive and anti-cancer activities [32,33]. Although curcumin has marked biological activity, its relatively poor solubility and stability in physiological conditions are well documented and represent its main drawbacks [33]. In order to increase the stability of curcumin, several curcumin-derivate were synthesized [34]. Curcumin and its analogues undergo keto-enol tautomerism. Concerning the antioxidant activity, the diketo form plays a key role in the regulation of redox homeostasis induced by curcumin [35], while the keto-enolic form shows a strong iron chelating ability [36]. Among Curcumin analogues, some β-diketo derivatives showed improved stability in physiological conditions, and increased antiproliferative activity against colon cancer cells HCT116 *in vitro* [33].

**Figure 1:**
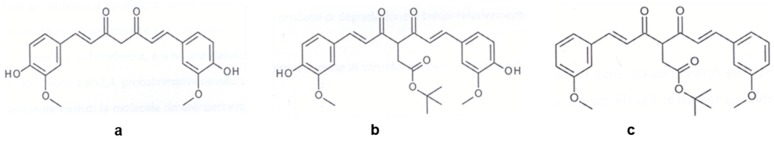
Structures of the investigated drugs loaded in the bioactive glasses: (**a**) curcumin; (**b**) K2T21; and (**c**) K2T23.

In the present contest, we report the study of the loading and release from bioactive glasses and mesoporous DDS of curcumin and two synthetic derivatives (Figure 1). The aim of the present study is to optimize the loading conditions in order to increase the amount of curcuminoid in the DDS, and investigate the released in physiological conditions.

## 2. Materials and Methods

### 2.1. Synthesis of Bioactive Glasses and Curcumininoids

**Figure 2:**
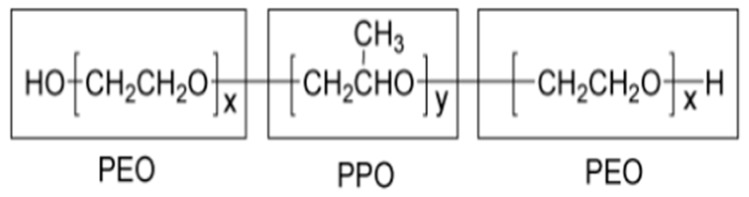
Structure of the self-assembling polymer Pluronic P123.

We synthesized a bioactive glass of composition 80SiO_2_∙15CaO∙5P_2_O_5_ (%mol) via sol-gel [37] and evaporation induced self-assembly (EISA) [38] methods. We obtained two samples with the same composition but different porosity: the sol-gel glass (hereafter as BG) was microporous, while the EISA glass (MBG) was mesoporous [30]. In both cases, we used tetra-ethyl-ortho-silicate (TEOS), calcium nitrate tetrahydrate and triethyl phosphate (TEP) as precursors for SiO_2_, CaO and P_2_O_5_ respectively. We used ethanol as solvent, HCl as catalyst and Pluronic P123 (Figure 2) as the self-assembling polymer in the EISA process. We ground the obtained glasses to a diameter <50 µm.

The *in vitro* bioactivity of BG and MBG was confirmed in previous papers [39,40]. In the present work, we want to evaluate the drug release both in pure distilled water and physiological environment, thus, we prepared a simulated body fluid (SBF) buffered at pH of 7.4, accordingly to Kokubo [41]. Table 1 shows the comparison between plasma and SBF ions concentrations (expressed as mM).

Curcumin, (3Z-5E)-*tert*-Butyl-4-hydroxy-6-(3-methoxy-4-hydroxyphenyl)-acrolyl)hexa3,5-dienoate (K2T21) and (3Z-5E)-*tert*-Butyl-4-hydroxy-6-(3-methoxyphenyl)-acrolyl)hexa3,5-dienoate (K2T23) (Figure 1a–c, respectively) were synthesized as previously reported [34].

### 2.2. Loading of Bioactive Glasses

We performed the loadings through a procedure called “evaporation”, and compared the new results on the loading with those obtained through immersion technique as reported in a previous paper [30]. The “evaporation method” can be summarized as follow: 100 mg of glass powder (diameter < 50 µm) were soaked in 5 mL of the curcuminoid ethanolic solution (1 mg/mL). After 30 min of stirring at room temperature (rt), we evaporated the solvent under *vacuum* to dry the glasses, then we put the glasses in the heater at 70 °C for 24 h. After drug loading, the samples were orange-yellow in color. The samples were characterized by elemental analysis, specific surface area (SSA) and porosity. The amount of curcuminoids were calculated with respect to blank samples, obtained by soaking the glass powder in ethanol, dryness under *vacuum* and treatment at 70 °C for 24 h.

### 2.3. Study of the Drug Release from Bioactive Glasses

We carried on static and dynamic release tests on the loaded samples. Briefly, static tests were performed as follows: loaded glass powder (50 mg) was suspended in distilled water (50 mL, glass/solvent ratio 1 mg/mL) in a PE bottle. The suspension was shaken in an orbital shaker (100 rpm) at 37 °C. At time intervals (5′, 15′, 30′, 1 h, 2 h, 3 h, 4 h and 24 h) the suspension was microfiltered (ø 0.45 µm) on cellulose filter paper, and the obtained solutions were analyzed by UV-Vis spectroscopy.

For the dynamic release tests, 250 mg of loaded glass were dissolved in the solvent medium (50 mL of pure distilled water or SBF, glass/solution ratio 5 mg/mL) in a PE bottle. The suspension was shaken in an orbital shaker (100 rpm) at 37 °C. At different times (1, 2, 3, 4, 6, 8, 10, 12, 16, 20 and 24 h), the solution was removed to be analyzed, then 50 mL of fresh solvent (pure distilled water or SBF) were added to re-fill the bottle. The extracted solution was micro-filtered (ø 0.45 µm cellulose filter paper) and analyzed by UV-Vis spectroscopy.

The concentration of curcuminoids in the solutions was calculated based on calibration curves (concentration range 0.5–100 µM) by spectrophotometric analysis.

### 2.4. Instrumental Analysis

Elemental analysis data were collected by a PerkinElmer 2400 CHNS analyzer, the results are expressed as % (*w*/*w*) of the drug with respect to the bioactive glass.

Specific surface area (SSA) and porosity were evaluated for MBG glass before and after soaking in ethanol solution and evaporation *under vacuum*. Measurements were performed by N_2_ adsorption at −196 °C using a Micromeritics ASAP 2020 porosimeter. For SSA determination, data were processed by employing the BET model. The BJH model was used to analyze mesopores size distribution, and the “t-plot” (statistical thickness method) was adopted to evaluate the presence of micropores [42,43]. For a review of the applied methods, see Gregg and Sing [44]. Before N_2_ adsorption measurements, all samples were outgassed at rt for 24 h (residual pressure: ~10^−3^ Torr).

The UV-Vis spectra of the solutions were collected using a Cary 100 UV-visible spectrophotometer in the 200–600 nm range.

## 3. Results

### 3.1. Characterization of the DDSs

Table 2 reports the percentages (*w*/*w*) of the drug loaded on the DDS (BG and MBG) using the “evaporation method”, data were calculated from the results of the CHNS analysis.

According to the applied experimental procedure, the maximum percentage of the loaded drug is 5% (*w*/*w*) (5 mg_(drug)_/100 mg_(glass)_). The data in Table 2 are below 5% for all curcuminoids and DDSs, hence the evaporation method allowed to load only 50% of the overall drug. If compared to the previous results [30], the amount of loaded drugs with the “evaporation methods” is ten times higher (0.3%/0.4% *vs.* 2%/4% *w*/*w*). With this method, part of the drug molecules ties to the glass pores through hydrogen bonds, while a portion remains in solution and precipitates on the surface during solvent evaporation. It is interesting to note that the MBG system is able to load a higher amount with respect to BG glass only in the case of curcumin, which is the smallest molecule. In addition, the amounts of curcumin and K2T21 loaded are higher with respect to K2T23 probably due to the presence of OH groups in the cited molecules, which are able to interact positively with the silanol (Si-OH) groups on the glass surface.

BET analysis (Table 3) confirms the reduced ability of MBG glass to host drug molecules in the pores.

The solvent clearly decrease the area of mesopores, where it should be possible to host the curcuminoid molecules.

### 3.2. Static Release Study

It is well known that curcuminoids, once in water, undergo keto-enol tautomerism, and the two tautomers have typical absorption maxima at different λ_max_: 300–380 nm for the diketo form (DK) and 400–470 nm for the keto-enol form (KE). In addition, the curcuminoids in solution originate products of degradation that absorb mainly in the range of the DK form and below (<300 nm) [45]. Preliminary analysis of the aqueous drug solutions were performed to select the absorption band to investigate. The analyses showed that in water:

(i) the DK form is the most stable one for all the investigated curcuminoids (~300–350 nm); and
(ii) degradation products show strong absorptions below 300 nm and in the range 300–350 nm.

**Figure 3:**
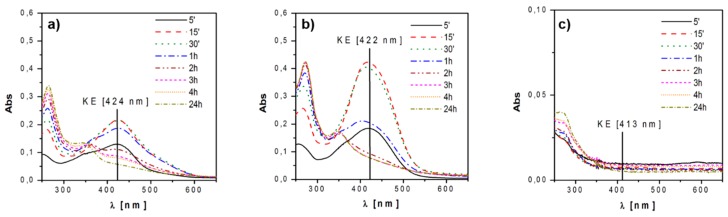
Static release tests performed in pure water at different times: (**a**) curcumin; (**b**) K2T21; and (**c**) K2T23.

In view of these remarks, we decided to survey and quantify the absorption of the band attributable to the KE form. Figure 3 shows the results of the static release tests in water. We observed that both curcumin and K2T21 behaved in a similar way: they were released by the glass in the KE form and as degradation byproducts. After 15/30 min, they started to convert into diketo form (DK) and degradation products; the latter are the principal components in solution after 24 h. The structure of the K2T23 is different from the two previous drugs because of the lack of hydroxyl groups, and thus, its behavior has been quite different with respect to the other release tests. This molecule is the less soluble and a very small fraction was released in pure distilled water, undergoing fast degradation. Indeed, solution obtained from the release tests of both curcumin and K2T21 showed an absorption band with a maximum at 424 and 422 nm, respectively, the range of the keto-enol (KE) form, while the solution obtained from the release of K2T23 absorbed only below 300 nm, in the range of the DK form and degradation products.

In this condition, the maximum release was reached after 30 min with a concentration of 7 and 43 µM for curcumin and K2T21, respectively. On the contrary, as previously reported, the concentration in water for K2T23 was negligible.

### 3.3. Dynamic Release Study

Since both the BG and the MBG glasses, when soaked in pure water with a ratio of 1 mg/mL released the drugs only for the first two hours, we decided to perform the dynamic tests soaking at 5 mg/mL glass/solution ratio. We soaked the same quantity of loaded glasses also for the tests in SBF. This solid/solution ratio was chosen because it is the same ratio used in bioactivity tests [46]. We performed the release tests over 24 h because after this time the releases were negligible for all the three drugs.

**Figure 4:**
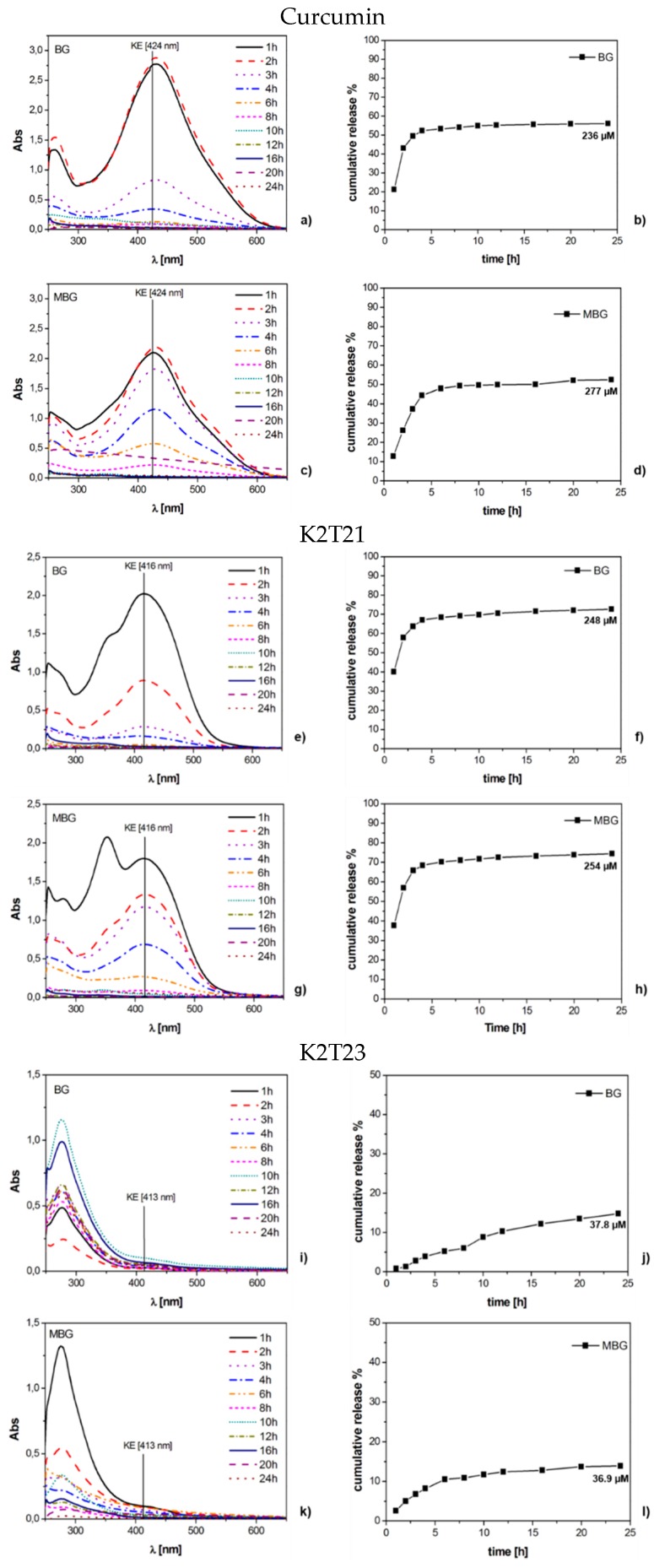
UV-Vis spectra and cumulative release in pure water (expressed in %) of BG and MBG samples loaded with: curcumin (**a**–**d**); K2T21 (**e**–**h**); and K2T23 (**i**–**l**).

In Figure 4 we reported the UV-Vis spectra of the experiments of dynamic release performed in pure water and the cumulative releases of the no degraded drugs, expressed in percentage. Curcumin (Figure 4a,c) and K2T21 (Figure 4e,g) behaved differently: curcumin was released as a mix of KE form and degradation products. K2T21, instead, was released as both KE and DK forms and degradation products. Thus, it is confirmed that the presence of the substituent in α position between the carbonyl and enol carbons shifts the equilibrium in favor of the DK form, increasing the stability in aqueous medium [34]. For K2T21, the tests performed over the BG and MBG did not show significant differences. Curcumin, instead, showed a lower and more controlled release from the MBG with respect to the BG. From the release curves, it is possible to notice that K2T21 was released in a higher quantity compared to curcumin, and this is strongly related to its higher stability in solution [34]. K2T23 (Figure 4i,k) always behaved in the same way, regardless of the glass or the concentration used. We detected only a very low signal of its KE form, while the most intense signals, below 350 nm, were the ones of its degradation products.

**Figure 5:**
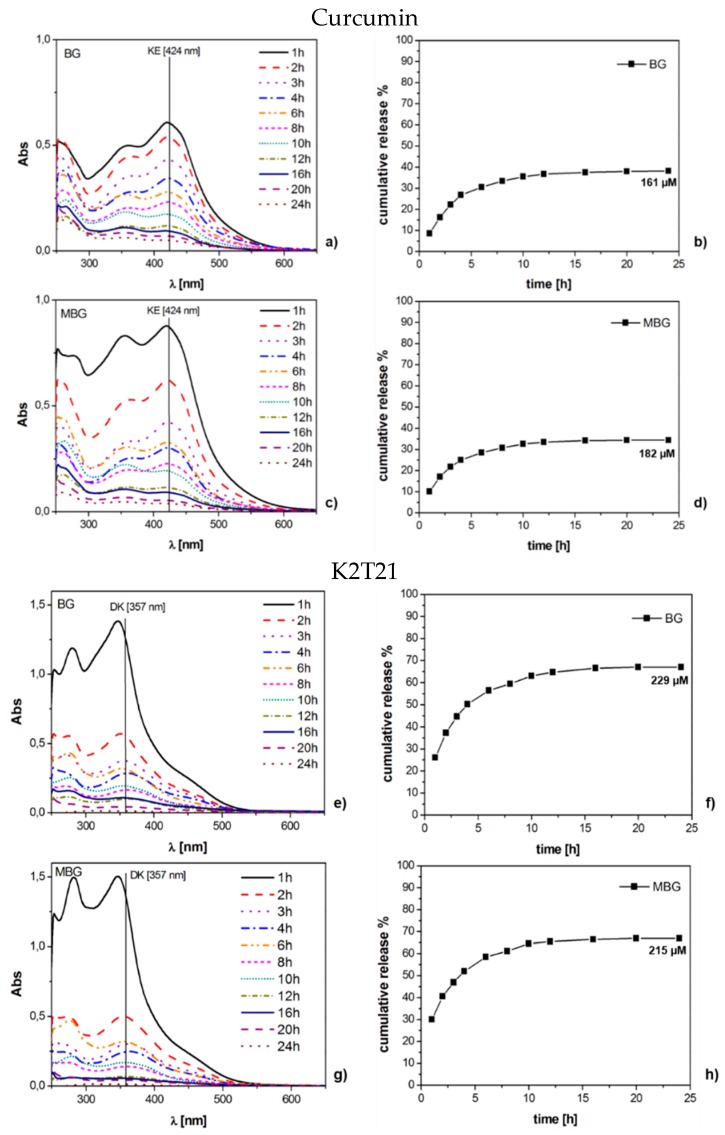
UV-Vis spectra and cumulative release in SBF (expressed in %) of BG and MBG samples loaded with: curcumin (**a**–**d**); and K2T21 (**e**–**h**).

**Figure 6:**
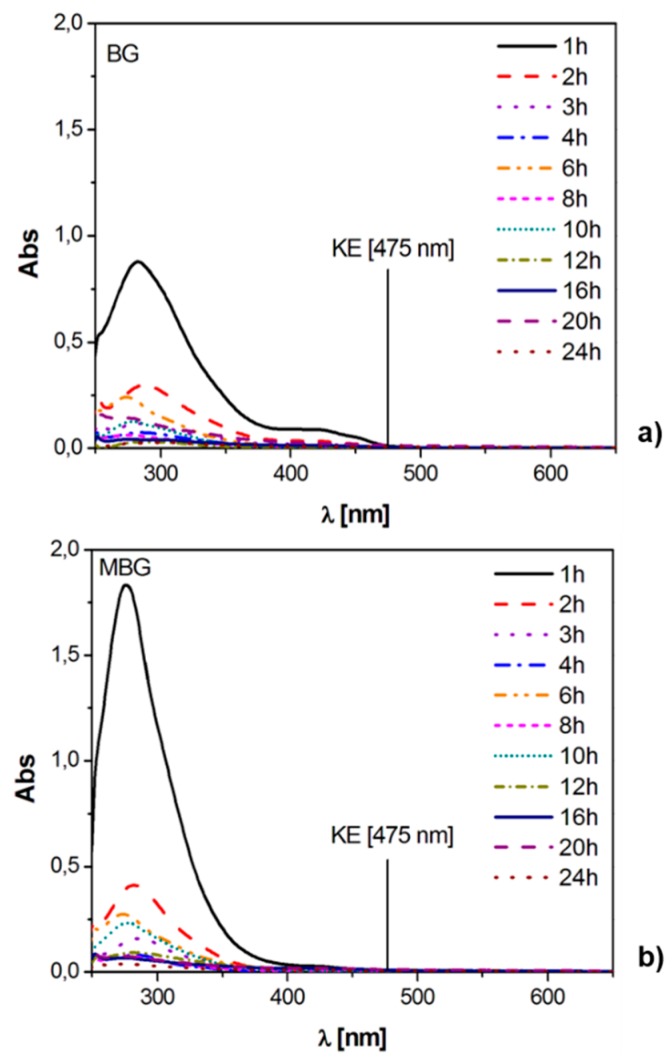
UV-Vis spectra in SBF of (**a**) BG and (**b**) MBG samples loaded with K2T23.

The UV-Vis spectra of the dynamic release tests performed in SBF are reported in Figure 5 (curcumin and K2T21) and Figure 6 (K2T23), together with the cumulative release expressed in percentage. K2T23 performed the same as in pure water; that is, all its spectra showed bands in the range of the degradation products. In SBF, the quantitation of K2T23 was prevented because this compound is completely released as byproducts. Regarding curcumin and K2T21, we could not compare how the two drugs behaved in SBF, as we did in pure water, but we noticed that in this environment, the substrate morphology (MBG *vs.* BG) did not really affect the releases. Spectra of curcumin releases showed three bands: the KE with maximum at 424 nm, the DK at 357 nm and one below 300 nm due to the degradation products (mainly vanillin [31]). All of the signals were quite intense at the beginning, then after 6–8 h of soaking the KE and the DK signals became much less intense than the band of the degradation products. The spectra of the solutions released from K2T21 showed a weak shoulder at 450 nm, attributable to the absorption of the KE form, and two intense bands at 357 nm (DK form) and below 300 nm (degradation products).

## 4. Discussion

The collected data highlight that all the drugs that we tested undergo degradation processes, both in water and SBF. Due to its very low aqueous solubility, K2T23 is not released in water or SBF as no degraded drug, and it is the most instable compound; indeed, we not detected signals of the KE form even after 5 min of soaking (Figure 3c). Further investigation on this drug can help to understand if these molecules degrade before the release, when they still lay on the glass, or as soon as they are released in solution. Furthermore, it could be interesting to figure out if also the degradation products of the K2T23 have anti-cancer properties, as the starting molecules. Regarding curcumin and K2T21, most of these molecules are released in the KE form, and then they converted in DK form and degradation products. When soaked in water, K2T21 is released as both KE and DK forms. Thus, the ester part of the molecule in position 4 increases the stability of the DK form. In water, during dynamic release, the concentration of K2T21 after 24 h was 250 µM, which represents almost the 70% of the loaded drug. The concentrations detected in SBF were slightly lower. Moreover, the difference of the stable form of the K2T21 in water (KE) and in SBF (DK) is due to the pH. SBF is a solution buffered at physiological pH (7.4), while the soakings in water were not buffered and the dissolution of the glass causes the increase of the pH. At acidic and physiological pH, the more stable form of the K2T21 is the DK, while at basic pH the more stable form is the KE [47].

For both curcumin and K2T21, the releases in SBF are a little bit lower than that collected in pure water. This happens likely because in SBF occurs the precipitation of hydroxyapatite (HAp) [24], which inhibits the release of the drug from the surface of the sample.

Regarding the case of curcumin, which is the smallest molecule, the drug could fit inside the mesoporous. Thus, a more controlled and slower release, with respect to the one of K2T21 and K2T23, was obtained (Figure 4d). It is interesting to note that when the drugs employed are curcumin and K2T21, both MG and MBG DDS are able to release not degraded drug molecules.

We tried to linearize the releases of the drugs through the Higuchi equation, as we already did in [30]. The linearizations were not possible, thus the mechanism of release is not attributable only to the diffusion.

## 5. Conclusions

It is possible to conclude that BG and MBG systems could be employed to deliver drugs that are highly instable in aqueous environments. The data collected to date are promising and encourage us to continue the study on the possibility to obtain drug delivery systems by coupling curcuminoids and bioactive glasses.

The ability of mesoporous-sol-gel (MBG) and sol-gel bioactive glasses (BG) to act as DDS was confirmed. In particular, Curcumin and K2T21 were released continually over 24 h in KE and DK form. However, with the increment of the time the amount of degraded drugs released were increased. The amount of loaded drugs depends on the dimension and the polarity (presence of –OH groups) of curcumin-derivates, while the release depends on the two previous factors and on the type of solution where the DDS was soaked.

## Figures and Tables

**Table 1:** Comparison between the composition (mM) of human plasma and simulate body fluid (SBF).

	Na^+^	K^+^	Ca^2+^	Mg^2+^	Cl^−^	HPO_4_^2−^	HCO^3−^	SO_4_^2−^
Plasma	142.0	5.0	2.5	1.5	103.0	1.0	27.0	0.5
SBF	142.0	5.7	2.5	1.5	147.8	1.0	4.2	0.5

**Table 2:** Percentage (*w*/*w*) of the loaded drug on the glass delivery system via evaporation method (SD = standard deviation obtained by three different replicate determinations).

Glass	Drug	%_drug_ (*w*/*w*) ± SD
BG	Curcumin	3.1 ± 0.1
	K2T21	3.3 ± 0.1
	K2T23	2.3 ± 0.2
MBG	Curcumin	3.9 ± 0.2
	K2T21	3.1 ± 0.1
	K2T23	2.4 ± 0.1

**Table 3:** N_2_ adsorption analysis performed on MBG sample before and after the soaking in ethanol.

	SSA (m^2^/g)	BJH Mesopore Area (m^2^/g)	BJH Average Pore Width (Å)
BG before	120	10	–
BG after	93	3	–
MBG before	472	224	30
MBG after	359	162	30

## References

[1] Van Gaalen S., Kruyt M., Meijer G., Mistry A., Mikos A., van den Beucken J., Jansen J., de Groot K., Cancedda R., Olivo C., van Blitterswijk C., Thomsen P., Lindahl A., Hubbell J., Williams D.F., Cancedda R., de Bruijn J.D., Sohier J. (2008). Chapter 19—Tissue engineering of bone. Tissue Engineering.

[2] Li Y., Liu T., Zheng J., Xu X. (2013). Glutaraldehyde-crosslinked chitosan/hydroxyapatite bone repair scaffold and its application as drug carrier for icariin. J. Appl. Polym. Sci..

[3] Mouriño V., Boccaccini A.R. (2010). Bone tissue engineering therapeutics: Controlled drug delivery in three-dimensional scaffolds. J. R. Soc. Interface.

[4] Lee H., Ahn S.-H., Kim G.H. (2012). Three-dimensional collagen/alginate hybrid scaffolds functionalized with a Drug Delivery System (DDS) for bone tissue regeneration. Chem. Mater..

[5] Vo T.N., Kasper F.K., Mikos A.G. (2012). Strategies for controlled delivery of growth factors and cells for bone regeneration. Adv. Drug Deliv. Rev..

[6] Kim H.-W., Knowles J.C., Kim H.-E. (2005). Hydroxyapatite porous scaffold engineered with biological polymer hybrid coating for antibiotic Vancomycin release. J. Mater. Sci. Mater. Med..

[7] Kim H.-W., Knowles J.C., Kim H.-E. (2005). Porous scaffolds of gelatin-hydroxyapatite nanocomposites obtained by biomimetic approach: Characterization and antibiotic drug release. J. Biomed. Mater. Res. B Appl. Biomater..

[8] Balcerzak J., Mucha M. (2010). Analysis of model drug release kinetics from complex matrices of polylactide-chitosan. Prog. Chem. Appl. Chitin.

[9] Tigani D., Zolezzi C., Trentani F., Ragaini A., Iafisco M., Manara S., Palazzo B., Roveri N. (2007). Controlled release of vancomycin from cross-linked gelatine. J. Mater. Sci. Mater. Med..

[10] Hum J., Boccaccini A.R. (2012). Bioactive glasses as carriers for bioactive molecules and therapeutic drugs: A review. J. Mater. Sci. Mater. Med..

[11] Domingues Z.R., Cortés M.E., Gomes T.A., Diniz H.F., Freitas C.S., Gomes J.B., Faria A.M.C., Sinisterra R.D. (2004). Bioactive glass as a drug delivery system of tetracycline and tetracycline associated with β-cyclodextrin. Biomaterials.

[12] Zhao L., Yan X., Zhou X., Zhou L., Wang H., Tang J., Yu C. (2008). Mesoporous bioactive glasses for controlled drug release. Microporous Mesoporous Mater..

[13] Garg T., Singh O., Arora S., Murthy R.S.R. (2012). Scaffold: A novel carrier for cell and drug delivery. Crit. Rev. Ther. Drug Carr. Syst..

[14] Mouriño V., Cattalini J.P., Roether J.A., Dubey P., Roy I., Boccaccini A.R. (2013). Composite polymer-bioceramic scaffolds with drug delivery capability for bone tissue engineering. Expert Opin. Drug Deliv..

[15] Rezwan K., Chen Q.Z., Blaker J.J., Boccaccini A.R. (2006). Biodegradable and bioactive porous polymer/inorganic composite scaffolds for bone tissue engineering. Biomaterials.

[16] Kim H.-W., Knowles J.C., Kim H.-E. (2004). Hydroxyapatite/poly(ε-caprolactone) composite coatings on hydroxyapatite porous bone scaffold for drug delivery. Biomaterials.

[17] Yaylaoğlu M.B., Korkusuz P., Örs Ü., Korkusuz F., Hasirci V. (1999). Development of a calcium phosphate-gelatin composite as a bone substitute and its use in drug release. Biomaterials.

[18] Zhang Y., Zhang M. (2002). Calcium phosphate/chitosan composite scaffolds for controlled *in vitro* antibiotic drug release. J. Biomed. Mater. Res..

[19] Kundu B., Lemos A., Soundrapandian C., Sen P.S., Datta S., Ferreira J.M.F., Basu D. (2010). Development of porous HAp and β-TCP scaffolds by starch consolidation with foaming method and drug-chitosan bilayered scaffold based drug delivery system. J. Mater. Sci. Mater. Med..

[20] Francis L., Meng D., Knowles J.C., Roy I., Boccaccini A.R. (2010). Multi-functional P(3HB) microsphere/45S5 Bioglass^®^-based composite scaffolds for bone tissue engineering. Acta Biomater..

[21] Li W., Nooeaid P., Roether J.A., Schubert D.W., Boccaccini A.R. (2014). Preparation and characterization of vancomycin releasing PHBV coated 45S5 Bioglass^®^-based glass–ceramic scaffolds for bone tissue engineering. J. Eur. Ceram. Soc..

[22] Yao Q., Nooeaid P., Roether J.A., Dong Y., Zhang Q., Boccaccini A.R. (2013). Bioglass^®^-based scaffolds incorporating polycaprolactone and chitosan coatings for controlled vancomycin delivery. Ceram. Int..

[23] Olalde B., Garmendia N., Sáez-Martínez V., Argarate N., Nooeaid P., Morin F., Boccaccini A.R. (2013). Multifunctional bioactive glass scaffolds coated with layers of poly(d,l-lactide-co-glycolide) and poly(n-isopropylacrylamide-co-acrylic acid) microgels loaded with vancomycin. Mater. Sci. Eng. C.

[24] López-Noriega A., Arcos D., Izquierdo-Barba I., Sakamoto Y., Terasaki O., Vallet-Regí M. (2006). Ordered mesoporous bioactive glasses for bone tissue regeneration. Chem. Mater..

[25] Xia W., Chang J. (2006). Well-ordered mesoporous bioactive glasses (MBG): A promising bioactive drug delivery system. J. Control. Release.

[26] Arcos D., Vallet-Regí M. (2010). Sol-gel silica-based biomaterials and bone tissue regeneration. Acta Biomater..

[27] Zhu M., Zhang L., He Q., Zhao J., Guo L., Shi J. (2011). Mesoporous bioactive glass-coated poly(l-lactic acid) scaffolds: A sustained antibiotic drug release system for bone repairing. J. Mater. Chem..

[28] Vallet-Regi M., Rámila A., del Real R.P., Pérez-Pariente J. (2001). A new property of MCM-41: Drug delivery system. Chem. Mater..

[29] Muñoz B., Rámila A., Pérez-Pariente J., Díaz I., Vallet-Regí M. (2003). MCM-41 organic modification as drug delivery rate regulator. Chem. Mater..

[30] Malavasi G., Ferrari E., Lusvardi G., Aina V., Fantini F., Morterra C., Pignedoli F., Saladini M., Menabue L. (2011). The role of coordination chemistry in the development of innovative gallium-based bioceramics: The case of curcumin. J. Mater. Chem..

[31] Shruti S., Salinas A.J., Ferrari E., Malavasi G., Lusvardi G., Doadrio A.L., Menabue L., Vallet-Regi M. (2013). Curcumin release from cerium, gallium and zinc containing mesoporous bioactive glasses. Microporous Mesoporous Mater..

[32] Hatcher H., Planalp R., Cho J., Torti F.M., Torti S.V. (2008). Curcumin: From ancient medicine to current clinical trials. Cell. Mol. Life Sci..

[33] Basile V., Ferrari E., Lazzari S., Belluti S., Pignedoli F., Imbriano C. (2009). Curcumin derivatives: Molecular basis of their anti-cancer activity. Biochem. Pharmacol..

[34] Ferrari E., Pignedoli F., Imbriano C., Marverti G., Basile V., Venturi E., Saladini M. (2011). Newly synthesized curcumin derivatives: Crosstalk between chemico-physical properties and biological activity. J. Med. Chem..

[35] Fujisawa S., Atsumi T., Ishihara M., Kadoma Y. (2004). Cytotoxicity, ROS-generation activity and radical-scavenging activity of curcumin and related compounds. Anticancer Res..

[36] Borsari M., Ferrari E., Grandi R., Saladini M. (2002). Curcuminoids as potential new iron-chelating agents: Spectroscopic, polarographic and potentiometric study on their Fe(III) complexing ability. Inorg. Chim. Acta.

[37] Hench L.L., West J.K. (1990). The sol-gel process. Chem. Rev..

[38] Brinker C.J., Lu Y., Sellinger A., Fan H. (1999). Evaporation-induced self-assembly: Nanostructures made easy. Adv. Mater..

[39] Shruti S., Salinas A.J., Malavasi G., Lusvardi G., Menabue L., Ferrara C., Mustarelli P., Vallet-Regì M. (2012). Structural and *in vitro* study of cerium, gallium and zinc containing sol-gel bioactive glasses. J. Mater. Chem..

[40] Salinas A.J., Shruti S., Malavasi G., Menabue L., Vallet-Regí M. (2011). Substitutions of cerium, gallium and zinc in ordered mesoporous bioactive glasses. Acta Biomater..

[41] Kokubo T., Kushitani H., Sakka S., Kitsugi T., Yamamuro T. (1990). Solutions able to reproduce *in vivo* surface-structure changes in bioactive glass-ceramic A-W3. J. Biomed. Mater. Res..

[42] Brunauer S., Emmett P.H., Teller E. (1938). Adsorption of gases in multimolecular layers. J. Am. Chem. Soc..

[43] Barrett E.P., Joyner L.G., Halenda P.P. (1951). The determination of pore volume and area distributions in porous substances. I. Computations from nitrogen isotherms. J. Am. Chem. Soc..

[44] Gregg S.J., Sing K.S.W. (1982). Adsorption, Surface Area and Porosity.

[45] Nardo L., Maspero A., Selva M., Bondani M., Palmisano G., Ferrari E., Saladini M. (2012). Excited-state dynamics of bis-dehydroxycurcumin carboxylic acid, a water-soluble derivative of the photosensitizer curcumin. J. Phys. Chem. A.

[46] Nicolini V., Varini E., Malavasi G., Menabue L., Menziani M.C., Lusvardi G., Pedone A., Benedetti F., Luches P. (2016). The effect of composition on structural, thermal, redox and bioactive properties of Ce-containing glasses. Mater. Des..

[47] Wang Y.-J., Pan M.-H., Cheng A.-L., Lin L.-I., Ho Y.-S., Hsieh C.-Y., Lin J.-K. (1997). Stability of curcumin in buffer solutions and characterization of its degradation products. J. Pharm. Biomed. Anal..

